# A Hospital Day in Salonika

**Published:** 1917-10-13

**Authors:** B. Holroyd Slater

**Affiliations:** formerly Medical Superintendent of the Bradford Workhouse.


### A HOSPITAL DAY IN SALONIKA.

By B. HOLROYD SLATER, M.D., formerly Medical Superintendent of the Bradford Workhouse.

We are indebted to the *Yorkshire Observer* for the following interesting account of the life of a medical man in a general hospital in the neighbourhood of Salonika:—

I rejoined my own unit on June 21, and since have had a glut of work. An equipment of tents, etc., was all dumped on to the site, and all hands set to work in levelling ground, pitching tents, making roads, etc., *ad infinitum*. In less than a week we had beds fully equipped for about 800 cases. Two days later we went up to 1,000 beds and had 900 patients. By Sunday night next—it is Friday now—we shall have 1,600 beds. We are all canvas, and very nice we look, spread out on the side of a hill crowned with great rocks, something like Brimham, and an old monastery thickly girt about with all manner of fruit trees. Further away, and just behind the officers' lines, is a precipitous crag where eagles have their homes. Fine fellows they are too! We are a good way out of Salonika, amongst the hills, and I'll back our site against that of any other hospital in this area. I'm told Salonika is an interesting old place. Maybe, but I don't want to explore it. For my sins and in the service of the Allied forces, I've had to go in pretty often of late. But the business in hand, the heat, dust, dirt, and stenches have made me too tired for archæological researches.

This is the life. I rise at 6, bathe under a cold tap, breakfast by 7.30, reach my office 7.45 to 8, and from this point onwards to 12.30 time flies, flies, and not a dull moment; 1 to 1.30 lunch; 1.30 to 2.30 is spent in a horizontal position in my tent. Then another mad rush by Father Time until 7. My great work is to see that everybody else in the surgical division whacks away at his or her work; advice and admonition are freely given at all times. The first is much in request, the latter often earned. Presently one suspects that routine will be established, and that by cutting out the admonitions I may secure some afternoons a week to my own private uses. I think you would like my tent, ordinary bell variety though it is. With the powerful aid of a hefty batman I've excavated the floor to a depth of 3 feet, more than 2½ feet of it through solid rock. This gives head-room all round. The support for the tent-hole is a portion of an ancient marble column—Greek no doubt, since we are in Greece. I dug it out of the side of the hill about 300 yards away. It is 4 feet high and 18 inches in diameter, weight probably half a ton. With a plank and levers we got it into a barrow and then found we couldn't lift the handles. So we outed it, and rolled it down the hill to my very door—superhuman efforts, worthy of a Pharaoh—and it now reposes in my tent, a source of wonder to generations yet unborn. The top of it, all round the pole, serves as an occasional table for bric-à-brac, such as a spare pipe and my shaving tackle.

The floor is covered with expensive rush mats—the Greek is a good business man. Red Cross packing-cases, ranged round on top of the parapet, give plenty of cupboard room, which is much appreciated by great and small of the ant family—a truly wonderful race; but the epic of our struggle for existence against one another would take too long to write. Besides, the end is not yet in sight, and the result is too uncertain, though I have hopes. Beetles—stout fellows 3 inches long, and a great horn as long again on their latter ends—beetles with and without wings, and of all the most striking colours—gorgeous butterflies, centipedes by the foot (my batman killed four in my temporary tent). There is "some" life here—I killed a snake in my tent a week ago; it is 4 feet long. One man here found one over 5 feet long curled up behind his books. . . . It's hot, of course, during the day, but not as bad as Egypt in summer. And the nights are delicious. My personal opinion is that Salonika is a much maligned place. This is really a health resort.

